# Supporting underrepresented students in health sciences: a fuzzy cognitive mapping approach to program evaluation

**DOI:** 10.1186/s12909-024-05292-7

**Published:** 2024-03-20

**Authors:** Danielle F. Chiang, Scott A. Guerrero, Emma C. Sexton, Stephen S. Gardner

**Affiliations:** 1 The University of Missouri, Institute for Human Development, 5306 Holmes Street, Kansas City, MO 64110 USA; 2 School of Medicine, The University of Missouri, 2411 Holmes Street, Kansas City, MO 64108 USA; 3 Children’s Mercy Hospital, 3101 Broadway Boulevard, Kansas City, MO 64111 USA

**Keywords:** Mentoring, Medicine education, Dentistry education, Pharmacy education, Health sciences, Fuzzy cognitive mapping, Mixed-methods, Student retention, Pipeline

## Abstract

**Background:**

The Students Training in Academia, Health, and Research (STAHR) Program at the University of Missouri-Kansas City (UMKC) strives to help students from low-income families that have experienced educational challenges due to poverty and prepare them to enter, persist, and graduate from a health sciences degree program at UMKC. Students in the program participated in fuzzy cognitive mapping (FCM) sessions to ensure that all voices of the program were heard to improve program implementation, and student success, and contribute to an equitable educational environment.

**Methods:**

Fuzzy Cognitive Mapping sessions for the 2020–2021 cohort of students (*n* = 52) were conducted to assess the strengths and weaknesses in program implementation, especially through the beginning of the COVID-19 pandemic. Students’ maps were coded by a team of researchers and then confirmed using confirmatory factor analysis.

**Results:**

Statistical analyses reveal that mentorship, workshops, and social support helped students to work toward their goal of obtaining a professional health sciences degree, while a lack of time, remote learning, and outside stressors inhibited their opportunities for success.

**Conclusions:**

The findings from a multipronged analysis of mapping data demonstrate the value of this innovative approach to the field, especially when looking to incorporate student voices.

**Supplementary Information:**

The online version contains supplementary material available at 10.1186/s12909-024-05292-7.

## Introduction

In Missouri and nationally, students from low-income families who experience educational challenges due to poverty enter and complete health professions degree programs at low rates. In a cross-sectional study of self-reported race/ethnicity of US medical school graduates from 2002 to 2017, “numbers and proportions of Black, Hispanic, and American Indian or Alaska Native medical school matriculants increased, but at a rate slower than their age-matched counterparts in the US population, resulting in increased underrepresentation” [1]. Further, students from lower socioeconomic backgrounds are less likely to persist through the first year of college, “less likely to persist to a bachelor’s degree or to have graduate degree aspirations” [2], and their aspirations specifically for pursuing advanced degrees in medicine or law are much lower than those of their higher socioeconomic peers [2]. Although individuals who enter college as a means of social mobility often achieve some upward trajectory, this does not negate the extraneous experiences and pressures that a student with a low-income will encounter [2]. These students, who are often the first in their families to attend college, may experience what researchers call a “cultural mismatch” with college environments that are designed with mostly middle-class students in mind; this can cause a sense of alienation and discomfort that impedes academic performance [3, 4]. When first-year college students have access to mentors and peer role models who support them to persist in their studies and connect them with necessary resources, the outcomes improve [3].

The Students Training in Academia, Health, and Research (STAHR) Program was established in 2019 to meet the needs of first-generation students, students from underrepresented socioeconomic backgrounds, and students at risk of not persisting to graduation. The program achieves this goal through structured pipeline programs, which include hands-on experiences, academic support and supplemental instruction, student psychosocial and emotional development, financial literacy training, and mentorship. Eligible students are those who come from educationally or economically disadvantaged backgrounds and are currently enrolled in a pre-professional or professional health sciences program at the university and apply to participate in the STAHR program at the beginning of each academic year. The Program focuses on supporting students by providing additional programming and curriculum to overcome social barriers, such as feelings of disconnectedness and unease with the larger collegiate and/or programmatic community. These barriers have become more imperative due to the COVID-19 pandemic. Globally, students have expressed high distress levels, low engagement, and confidence in their academic performance and professional development in the wake of the COVID-19 pandemic [5, 6].

To identify aspects of the STAHR Program that best facilitate students’ success from the perspective of the students themselves, we utilized Fuzzy Cognitive Mapping (FCM) as a new approach to analyzing barriers for under-resourced students in health sciences, whose needs are often overshadowed by other priorities, further leading to cultural mismatch [4]. FCM is an evidence-based methodology proven as a reliable knowledge-based model that facilitates “sense-making” by helping program participants communicate strategies and decisions [7, 8]. Rather than traditional semi-structured interviews or focus groups, where information is collected qualitatively. FCM has its uniqueness in including a causal relationship in the approach, where participants provide quantification for connections based on their perception of the cause-and-effect relationships among concepts [9].

Fuzzy Cognitive Maps (FCM) were introduced by Kosko [8] as a soft method to represent causal reasoning in systems. FCMs are essentially a graph structure with nodes that represent important components in a system and edges that connect nodes to indicate the direction of the relationship between components. Each edge has a magnitude to indicate the strength of the relationship between nodes. The FCM analytical method permits qualitative graphs to be computable and analyzed using statistical techniques [7]. There are two general methods for constructing FCMs that can be combined in a hybrid fashion: expert-driven and data-driven [10]. Expert-driven FCMs use guidance and expertise provided by experts in the domain being analyzed to construct an FCM graph structure. Data-driven FCMs use data available on the system being analyzed to construct the FCM graph structure. Initially, FCM models were mostly constructed using expert-driven methods as data-driven methods took some time to develop [11, 12]. This study used an expert-driven approach to create the maps, with students in the program being the experts.

FCM helps us capture the intuitive knowledge of the students in the program and helps develop a multi-layered understanding of the critical components and processes within the program that support students to become successful in their program. FCM has been applied in a vast array of fields, such as improving agricultural policy design [13], assisting business leaders with their strategic management functions [14], knowledge sharing in urban planning [15], and understanding students’ quality of interaction within online learning in higher education [16]. Overall, FCM takes what Olazabal et al. describe as a “semi-quantitative” approach to describing complex systems by aggregating qualitative and quantitative data collected from mapping participants and combining the data in a way that permits an understanding of the system as a whole [17].

To demonstrate the FCM methodology and its usefulness in program evaluation efforts, this paper overviewed the FCM study conducted through idea mapping sessions with the 2020–2021 cohort of STAHR Ambassador students as part of the program evaluation process and demonstrated the supports and barriers identified by participants.

## Methodology

### Procedure

The research team conducted 13 virtual mapping sessions with 52 STAHR students over three weeks during the month of April 2021 via Zoom [18]. Each session consists of three to five students working independently to complete the map and then share out. In preparation, all researchers were adequately trained to facilitate mapping sessions with students. Additionally, team members were trained in coding and analysis procedures to ensure consistency throughout the evaluation process.

**Fig. 1:**
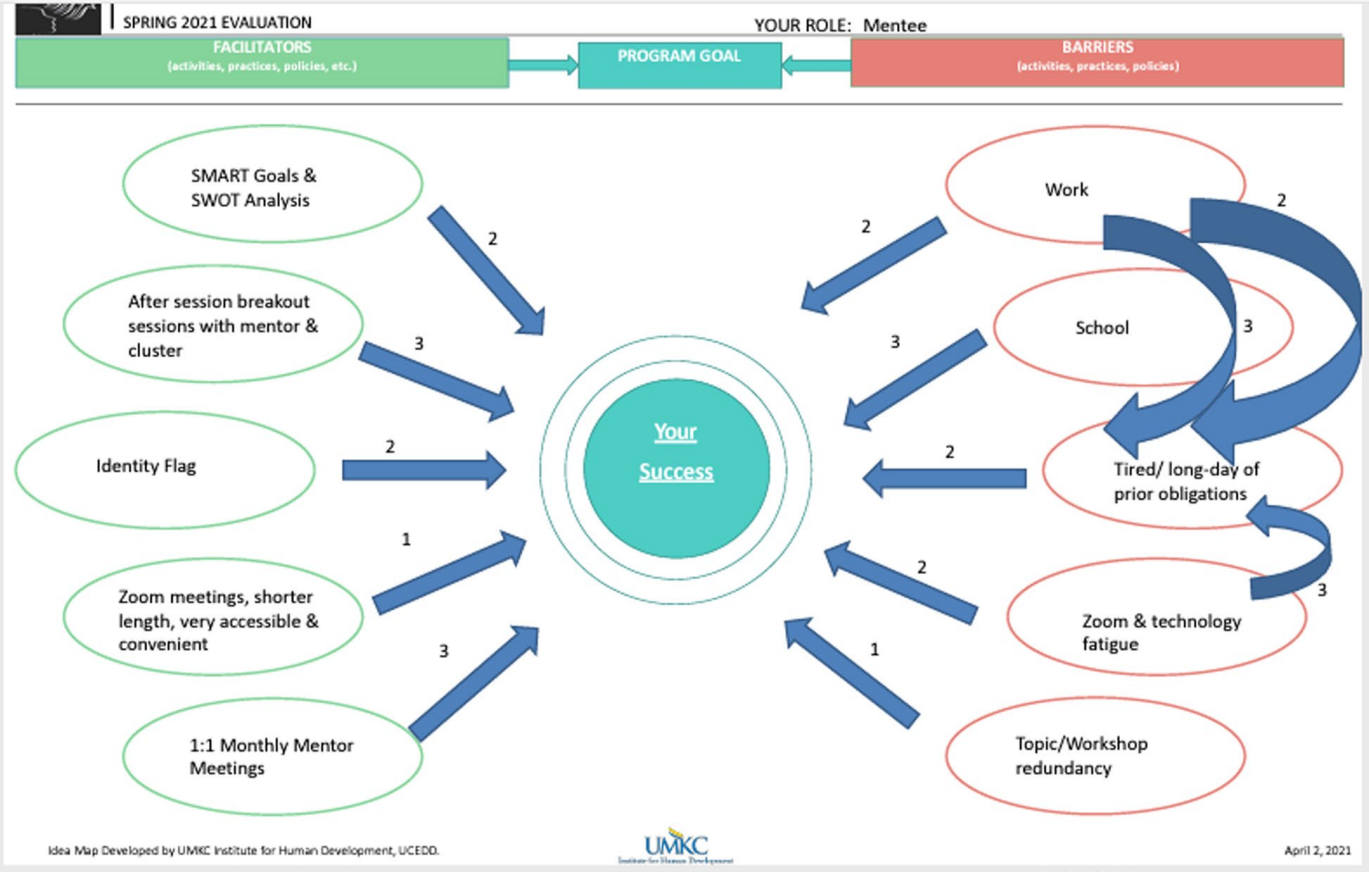
A sample concept map used in fuzzy cognitive mapping sessions for STAHR Ambassador Program evaluation at the University of Missouri-Kansas City, 2021

Each mapping session was facilitated by two researchers; at the beginning of each session, students were presented with a blank map. The blank map displays a center circle as the program goal, five ovals on the left side for facilitators to the goal, and five ovals on the right side for barriers to the goal. Before the mapping sessions began, students were assured that their responses would be held confidential and no identifiable information would be shared with the Program. First, we reminded students that the goal of STAHR is to ensure that students in the Program successfully complete their health science degrees, which is the center or destination of the map. Second, we asked students to fill three to five ovals on the left side of the map with activities, practices, or policies within the STAHR Program that facilitate the attainment of the program goal. Third, we asked students to fill three and five ovals on the right side of the map with activities, practices, or policies within or outside of the STAHR Program that create a barrier to the attainment of the program goal. Activities, practices, or policies that are identified as facilitating STAHR program goals are considered to have a positive causal impact, while those identified as barriers are considered to have a negative impact. Fourth, students drew directional arrows between ovals representing a connection between concepts and the overall goal. Finally, students rated the strength of each connection using a scale from one to three, with “1” being a weak connection, “2” being a moderate connection, and “3” being a strong connection. Figure 1 is an example of a completed map.

### Participants

A total of 52 students participated in mapping sessions in the Spring of 2021. Of the participating students, 59.6% (*n* = 31) enrolled in the medical program, 25.0% (*n* = 13) in the pharmacy program, and 15.4% (*n* = 8) in the dental program. Most participants were female (80.8%, *n* = 42) and reported that they were citizens of the United States (92.3%, *n* = 48). Almost forty percent (40.4%, *n* = 21) were Black/African American, 25.0% were Asian (*n* = 13), 19.2% were Caucasian (*n* = 10), 7.7% were multiracial (*n* = 4), and 7.7% reported their race as “other” (*n* = 4). Additionally, 21.2% of students (*n* = 11) self-identified as Hispanic or Latinx. Half of the students reported that English was the primary language spoken at home (*n* = 26), and 32.7% of students reported that they spoke a language other than English (*n* = 16), or a mix of English and another language (*n* = 1), when at home. All students reported being full-time students, and 11 students (21.2%) reported part-time employment. Table 1 provides the demographic information of the students.

**Table 1:** Participant Demographics; STAHR Ambassador Students (*n* = 52) at the University of Missouri-Kansas City, 2021

	**Students in Mapping Sessions (** ***n*** ** = 52)**	
	*n*	*%*
***School Affiliation***		
Dentistry	8	15.4
Pharmacy	13	25
Medicine	31	59.6
***Gender***		
Female	42	80.8
Male	10	19.2
***Race***		
Asian or Asian American	13	25
Black or African American	21	40.4
White or Caucasian	10	19.2
Other	4	7.7
More than one	4	7.7
**Ethnicity**		
Hispanic or Latinx	11	21.2
Non-Hispanic or Latinx	41	78.8
**Citizenship**		
U.S Citizen	48	92.3
Non-U.S Citizen	4	7.7
**Is English your primary language spoken in your home?**		
Yes	26	50
No	16	30.8
English and other language(s)	1	1.9
Not Reported	9	17.3
**Student Status**		
Full-time Student	51	98.1
Part-time Student	1	1.9
**Employment Status**		
Full-time Employment	0	0
Part-time Employment	11	21.2

Institutional Review Board approval for the study was secured through the university. Written informed consent was provided and obtained from students before each mapping session.

### Analysis

#### Coding

**Fig. 2:**
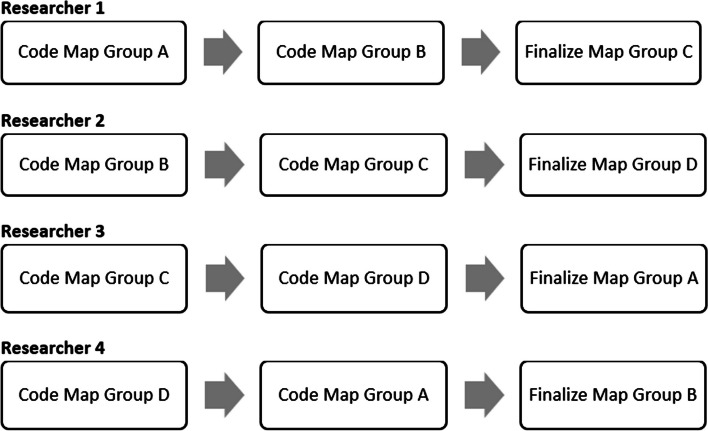
A flow chart of the coding process used in coding the 52 maps generated by students during the fuzzy cognitive mapping sessions at the University of Missouri-Kansas City, 2021

The research team started with item-level analysis, examining the concepts (i.e., facilitators and barriers) on the maps. Four team members carefully read through the maps, listened to the session audios, referred to the notes, and developed a codebook based on relevant and reoccurring themes found in students’ responses on the ovals (see codebook in Additional file 1). Together and using an inductive process, the research team finalized nine codes for facilitators and eight codes for barriers. Once the codebook was agreed upon by all four researchers, the 52 total maps were divided among four researchers. Each researcher blindly coded two groups of maps based on the codebook and finalized the third. The four researchers then got together to finalize the codes for all the maps, discussed any discrepancies, and reached a consensus on the codes assigned to each map. Audio recordings and notes were used in the process. Figure 2 displays the flowchart of the map coding process.

#### Statistical analysis

Following the coding process, we used Microsoft Excel [19] to create an adjacency matrix for each map. An adjacency matrix is a matrix that represents a network-type diagram or graph, directed (with arrows) or non-directed (without arrows) [8]. We used the directionality of the connecting arrows and the strength score assigned to each connection to develop each adjacency matrix. For better results in the analysis and easier interpretation, we normalized mapping data using the min–max normalization technique to put the strength scores in a range between 0 and 1. Specifically, raw scores of 1, 2, and 3 were translated to 0.25, 0.5, and 0.75 respectively. Under the fuzzy logic theory framework, students in the FCM sessions are experts with knowledge and experience with the STAHR program, we employed an expert-driven FCM approach, combining the map from each student to construct the final adjacency matrix following the process described by Kosko [8] and Stach et al. [11]. The final combined adjacency matrix was then exported to an Excel-based program called FCMapper (beta version, https://www.fcmappers.net/joomla/) for further analysis. Centrality is the key measure from FCMapper results and is calculated from the final combined adjacency matrix (a square matrix), where the i-th concept’s measure of centrality is found by summing the absolute values of elements from the i-th row and i-th column. Centrality is a measure of the importance of map concepts; therefore, concepts with high centrality indicate a high impact. The formula for centrality we used is based on Mago [20]:

$${{\text{C}}}_{{\text{i}}}=\sum \left|{{\text{A}}}_{{{\text{i}}}_{.}}\right|+\sum \left|{{\text{A}}}_{{.}_{{\text{i}}}}\right|$$

Where C_i_ is the measure of centrality for the i-th concept, A_i_. is the i-th row of the final combined adjacency matrix, and A._i_ is the i-th column of the final combined adjacency matrix.

Although FCMapper is helpful in investigating the network of responses using a single data set, this process does not confirm or validate the network structure using a statistical model. Therefore, using the same data, we employed confirmatory factor analysis (CFA) to confirm the underlying structure of the mapping data. As a part of the Structural Equation Modeling (SEM) family, CFA plays an essential role in model validation [21, 22]. CFA helps us understand the relationships between the mapping concepts and the underlying structure for supporting our students’ success in professional-level health degree programs. Analyses are conducted in R [23] and lavaan package [24]. An additional file showing the CFA R code is attached (see Additional file 2).

#### CFA model fit

Several model fit indices were used to determine if a CFA model fit well with the existing data. Chi-square statistic and Standardized Root Mean Residual (SRMR) were also used as one of the absolute fit statistics (SRMR < 0.08); SRMR represents the standardized difference between the observed and model-implied variances and covariances [25, 26]. In addition, Root Mean Squared Error of Approximation (RMSEA) is commonly used for absolute fit. It compares model fit to a perfect model and is expected to be smaller than 0.05. We also used a Comparative Fit Index (CFI), which indicates a close fit (CFI > 0.95), and Tucker-Lewis Index (TLI), which denotes a good fit (TLI > 0.95) to best prioritize the needs of STAHR students [25, 27, 28]. The model fit results from this analysis meet the threshold guidelines as outlined in Hu & Bentler and Jackson et al. [29, 30].

## Results

### FCMapper Results

A total of 52 maps were completed by students in the STAHR Program. We used 17 broad themes to code the maps. Nine of these concepts “facilitate” the program goal, meaning that they increase the likelihood that students will be successful in completing a professional health sciences degree and in the program. Eight of these concepts were “barriers,” meaning that they decrease the likelihood that students will be successful in completing their professional health sciences degree or in the program. There was one program structure-related concept that appeared both as a facilitator and a barrier, but with different aspects mentioned by students (see Appendix 1). As the program refines its structure from year to year based on students’ voices, more problematic program structure aspects will be resolved.

**Fig. 3:**
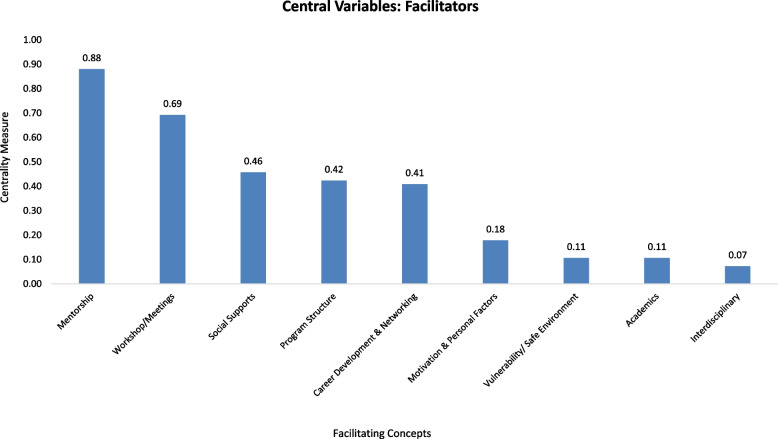
Concepts that facilitate students’ success in their health science degree and their corresponding centrality measure, as calculated during the FCM mapping study conducted at the University of Missouri-Kansas City in 2021

**Fig. 4:**
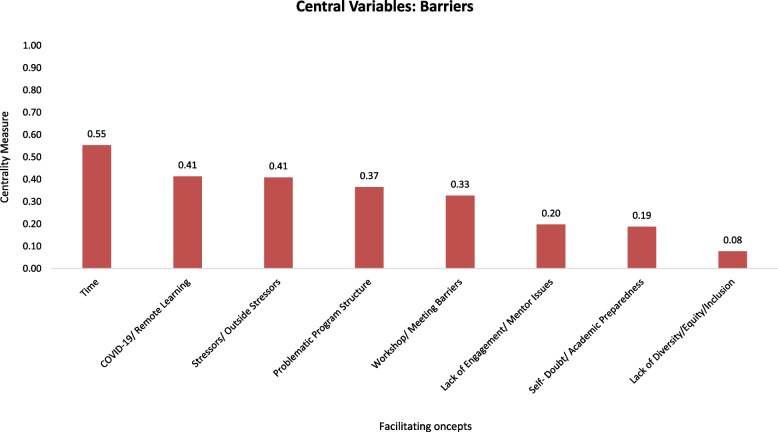
Concepts that inhibit students’ success in their health science degree and their corresponding centrality measure, as calculated during the FCM mapping study conducted at the University of Missouri-Kansas City in 2021

The facilitator concepts were ordered by their centrality (see Fig. 3). The net influence of a variable in a cognitive map can be understood by calculating its centrality (C), which shows how connected the variable is to other variables and the cumulative strength/weights of these connections. When using the fuzzy cognitive mapping technique, a variable can be more “central” although it has fewer connections if the connections carry larger weights or are stronger [8]. Essentially, “the centrality of the variable is not only a frequency of expression but also [exemplifies] how important that variable is given the whole structure of the cognitive map” [31]. In a similar fashion, “barrier” concepts were ordered by their centrality, as seen in Fig. 4. The centrality for each concept was calculated as a sum of the absolute values of edges’ weights connecting to a given concept as in Mago et al. [20].

To better interpret the results, we identified the top three facilitating concepts and the top three barrier concepts captured by students’ cognitive maps. The most impactful facilitator to student success was *“Mentorship*” (*n* = 46, C = 0.88), characterized by mentorship experiences involving both faculty and students. Students shared experiences that benefited them throughout the year such as being matched with a mentor within the same field of study, one-on-one meetings with mentors, and an overall sense of compassion and encouragement from their mentor. The second most impactful facilitating concept was “*Workshop/Meetings”* (*n* = 45, C = 0.69)*.* Students expressed that workshop topics, such as monthly STAHR meetings with different professionals who gave refreshing perspectives, were helpful to their career and professional development. The third most impactful facilitator was “*Social Supports*” (*n* = 26, C = 0.46), in which students identified facilitating concepts such as feeling a sense of belonging with other peers in the STAHR Program, support from friends and family, and welcoming STAHR staff to guide them through the challenges of being a first-generation or minoritized student.

Conversely, the most impactful barrier to success in the STAHR Program was “*Time*” (*n* = 31, C = 0.55), which included experiences such as overlapping and conflicting schedules between class or clinical schedules and programmatic requirements, as well as balancing and managing their time as a graduate-level student. The second and third most impactful barriers were “*COVID-19/Remote Learning*” (*n* = 29, C = 0.41) and “*Stressors/Outside Stressors*” (*n* = 23, C = 0.41); these concepts ranked equally in responses. Regarding “*COVID-19/Remote Learning,*” students shared that the decrease in activities due to COVID, Zoom/technology fatigue, and fewer opportunities for clinical experience hindered their capacity for success. For the “*Stressors/Outside Stressors*” concept, students expressed those tensions among balancing school, their personal lives, familial expectations, financial responsibilities, health issues, and feelings of homesickness were sometimes too burdensome, especially as low-income, educationally challenged, or first-generation students who often carry more external pressures than their peers.

### CFA results

**Fig. 5:**
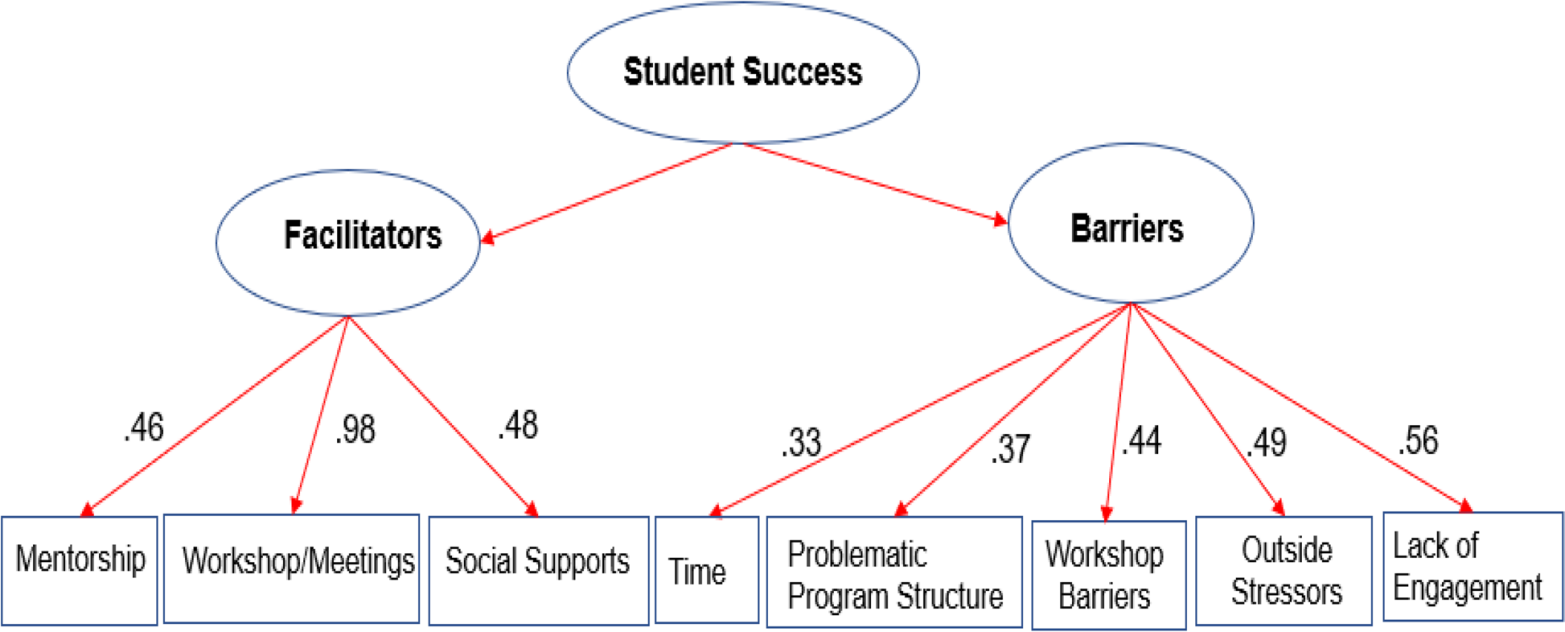
The most impactful facilitators and barriers to student success after conducting a CFA two-factor model analysis, as calculated during the FCM mapping study conducted at the University of Missouri-Kansas City in 2021

We conducted CFA (see code attached as Appendix 2) with the weights and connections on the maps to investigate the variables that facilitate or hinder student success. The two-factor model, with the “facilitator” and “barrier” as its individual latent variables, had an excellent fit, χ^2^(19) = 19.73, *p* = 0.41, CFI = 0.98, TLI = 0.97, RMSEA = 0.03, SRMR = 0.08. These results indicated that the top three impactful facilitating variables were 1) *Workshop/Meetings* (λ = 0.80), 2) *Social Supports* (λ = 0.54), and 3) *Mentorship* (λ = 0.50), which is consistent with the above-mentioned results. Five variables were retained in the “barriers” factor, with the top three being 1) *Lack of Engagement* (λ = 0.56), 2) *Outside Stressors* (λ = 0.49), and 3) *Workshop/Meeting Barriers* (λ = 0.44). Overall, the CFA analysis results are congruent with the results from FCMapper software (see Fig. 5). Figure 5 displays the factor loadings of variables to the construct. The larger the factor loading is, the more impact a variable has on the construct. Overall, combining the results from FCMapper and the CFA, in addition to the other concepts identified by FCMapper, we added “*Lack of Engagement*” to our final list of significant barriers in the STAHR program. Students who mentioned “*Lack of Engagement*” shared feedback about lacking one-on-one opportunities for mentorship, networking, or bonding with mentors.

## Discussion

### What matters to students?

#### Mentorship

The FCM approach helped the program understand the urgent needs of students from low-income families experiencing educational challenges due to poverty. The results indicated a few critical factors that contribute to or hinder STAHR students’ success in progressing from one year to another, obtaining a health sciences degree, and having a career in the field. As found in other research studies, many students from all walks of life find mentorship helpful, whether it’s from faculty or upper-level students [32]. The STAHR Program encourages students to have in-person meetings with their mentors at least once per month, if not more frequently, and to have additional virtual meetings if feasible. Based on the mentor–mentee encounter data reported by students during this cohort, a majority of their encounters (81.4% out of 117 encounters) were virtual (via Zoom, Teams, or phone). Topics for discussion included academic/professional development, psychological development, identity development, social development, and leadership development. Over 20 professional mentors served as direct support or liaison for their mentees in relation to these support areas. In that sense, it is imperative that the mentors are adequately skilled to ensure that they are able to meet their mentees where they are at, build a trusting relationship early on, and tailor support to the individual’s needs. In turn, it is also essential to assess and understand the well-being of the mentors to ensure that mentor–mentee relationships remain a productive priority within the program.

#### Stressors

Conversely, students experience stressors that hinder their success in the STAHR Program and within their health sciences degree. Time management is challenging for STAHR students, as they have to juggle between coursework, clinical rotations/shadowing, and employment, unlike many of their peers who do not need to rely on their own employment to finance their education [2]. In addition, students sometimes find themselves in a difficult situation where they are worn out from meeting virtually but are also unable to attend the in-person meetings because of their schedule. It is important for a program to understand the context it exists in and strike a balance between virtual meetings and in-person activities (e.g., workshops); our study shows that students appreciate when a program makes an effort to accommodate their schedules. Likewise, students reported experiencing financial difficulties. Given the social background of STAHR students, it is important for the program to understand that these students might be working hard to make ends meet for themselves and possibly for their entire family. As a result, students face additional stress in balancing their numerous conflicting priorities while participating in all program activities, which illuminates the need for additional support surrounding time management and workshop participation. Acknowledging this reality, the STAHR Program provides scholarships to students who actively participate in the program so that their financial stress may be lessened.

Furthermore, many of the barriers and stressors students experienced can also be attributed to the COVID-19 remote learning environment that caused feelings of a lack of engagement between students and the program. Prior to the COVID-19 pandemic, the racially and ethnically diverse students served by the STAHR program relied heavily on support services With little-to-no access to these support services, these students faced compounded challenges during the pandemic, whereas the traditional higher education environment was not equipped to respond in such times. For instance, due to a lack of readily available resources, students’ mental health was negatively affected, thus interrupting their academic performance. In addition, students experienced a heightened sense of financial insecurity as many students lost their jobs with no way to support themselves with basic needs like food and housing. The STAHR Program aimed to combat the challenges students were facing in and outside of the online classroom.

As students adapt to a new mode of learning, it is still important for support to be in place to care for students’ mental health, monitor their anxiety and stress levels, and make sure that psychological/social supports are provided on time. Students need person-centered, holistic support; it is unrealistic to assume the only support they will require when obtaining a health sciences degree is purely academic. For example, studies show that students benefit by receiving support in developing a sense of belonging to the professional field, as well as support in developing their professional identity [33]. The STAHR Program provided students with workshops that centered on strengthening students’ emotional and intellectual foundations so that they may be equipped to face various challenges in and outside of the classroom.

### Implications for incorporating student voices

The present study employed the fuzzy cognitive mapping technique as an exemplary tool for researchers and evaluators alike who aim to seek out the voices of those with lived experiences in a program to better understand the facilitators and inhibitors of program or participant success. In the field, there is always a struggle to provide inclusive and equitable support to students from underrepresented backgrounds. It is invaluable for a program to take into consideration students’ real needs (voices from students) and make modifications to tailor its support for future cohorts from existing cohorts, with an aspirational goal of making the program dynamic enough to adjust support for each student as they move through the curriculum. By doing so, the program promotes increased involvement and retention of students, which can then lead to better program outcomes.

Utilizing FCM allows us to analyze program successes and limitations from the students’ perspectives, thus creating a program tailored to students’ needs from year to year. Our findings highlighted the strengths of the STAHR program, indicating the structural elements that play integral roles in the success of students’ experiences. Through the mapping sessions, students in STAHR acknowledge that mentorship is the top facilitator of our program. Therefore, the program is more strategic in the matching of mentors and mentees. At the basic level, the program pairs each student with a professional mentor who practices in the same field in which the student is studying. Realizing how strong of a connection mentoring is, the program is able to go beyond simply aligning the health professions of both the mentor and the mentee. In analyzing the FCM data, the program has added questions to the program application and evaluation surveys to take a deeper dive into students’ life experiences. Additionally, the program inquires about students’ personality traits and interests such as hobbies and professional goals. This is done so the program can ensure students are matched with the best possible mentor we can provide. Since we know representation matters, leading to an increased sense of belonging [34], we strive to pair participants with mentors who possess similar background characteristics such as similar racial and ethnic groups, first-generation status, socioeconomic status, geographic origin, etc.

Further, the FCM results clearly demonstrated the need for why student voices are vital to their personal success as well as to the overall success of the STAHR Program. During the mapping sessions, students were grouped with 3–5 of their peers. Since relationships have already been established among participants, these sessions can exist as a safe place for students to share openly their thoughts and feelings about STAHR and their academic programs. Research indicated that students who feel they have a voice in class are seven times more likely to feel motivated than those who do not [35]. With all the rigors that come with being enrolled in a health professions program, students may not always have the space nor the “know-how” to assert their opinions as they relate to their learning. The STAHR Program aims to consistently provide spaces, whether through mentoring meetings, workshops, or mapping sessions, where students can authentically share their lived experiences. In doing so, the program is able to effectively change the course to better set students up for success for years to come.

### Limitations and future research

First of all, this study used a 3-point scale for simplicity. As we continue to use the FCM approach to incorporate students’ voices into program improvement, we will consider using a larger scale (e.g., a 5-point scale) to better capture the magnitude of casual relationships students provided to each concept on the maps. In addition, the data collection method was limited to Zoom due to the pandemic. Future research will explore the advantages and disadvantages of conducting mapping sessions on Zoom versus in person, as well as how that impacts students’ responses and experiences in the mapping sessions. One limitation of the study is that program outcomes were not included in the study. Given the scope of this study, factors directly related to outcomes were not investigated. Future research will focus on how the support students receive contributes to program outcomes, including academic performance, program, and school retention, post-graduation employment, etc. Future research will also examine the correlations between concepts (facilitators and barriers) in addition to the pathways to the center/goal. As the sample size increases, a more rigorous analysis with a full model of variables will provide a deeper understanding of the factors that impact students’ success.

Another limitation of the present study was the sample size; however, in the future, we hope to increase the sample size and examine the results on the subgroup level, based on race, ethnicity, gender, income level, and any other identifying factors that may lend itself to useful analysis. In order to better understand whether student needs vary by demographics and to create programs that are suited to these students' needs, it is advantageous to increase the sample size in order to complete analyses at the subgroup level. Another challenge is finding diverse mentors that match the race, ethnicity, and gender of the increased number of students in the program. Currently, mentors are university faculty and practitioners from partnering health institutions. With a growing need for more mentors, we will continue to recruit diverse professionals from local community hospitals. This could potentially be more challenging post-COVID with decreased numbers of available faculties and community professionals due to burnout.

## Conclusion

Fuzzy cognitive mapping offers researchers a multi-pronged approach that computes suggestions for improvement while allowing for aspects of programs that work to remain intact. Our study found that through the 2020–2021 academic year, the most helpful factors the STAHR Program provided were mentorship from peers and mentors in the community, workshops aimed to support their academic growth, and social support from the program and from their community. Alternatively, students struggled with balancing time between school and other programmatic obligations, being in a remote environment due to COVID-19, dealing with outside stressors, a program structure reflective of the program’s infancy, and misdirected workshop topics. In turn, the barriers identified by students were particularly valuable in pointing out the blind spots in the program implementation and directing areas for future program evolvement.

### Supplementary Information

**Additional file 1. **STAHR Ambassador Map Code Book; the codebook used by researchers to code maps created by STAHR students.

**Additional file 2. **Confirmatory Factor Analysis R Code.; the code used by the researcher to confirm the final two-factor structure of the concepts brought up in the maps by STAHR students.

## Data Availability

The datasets used and/or analyzed during the current study are available from the corresponding author upon reasonable request.

## Abbreviations

C: Centrality
CFA: Confirmatory Factor Analysis
CFI: Comparative Fit Index
FCM: Fuzzy Cognitive Mapping
RMSEA: Root Mean Squared Error of Approximation
SEM: Structural Equation Modeling
SRMR: Standardized Root Mean Residual
STAHR: Students in Training in Academia, Health, and Research
TLI: Tucker-Lewis Index
UMKC: University of Missouri-Kansas City
