# Correction: Supporting underrepresented students in health sciences: a fuzzy cognitive mapping approach to program evaluation

**DOI:** 10.1186/s12909-024-05360-y

**Published:** 2024-04-02

**Authors:** Danielle F. Chiang, Scott A. Guerrero, Emma C. Sexton, Stephen S. Gardner

**Affiliations:** 1 Institute for Human Development, The University of Missouri, 5306 Holmes Street, 64110 Kansas City, MO USA; 2 School of Medicine, The University of Missouri, 2411 Holmes Street, 64108 Kansas City, MO USA; 3 Children’s Mercy Hospital, 3101 Broadway Boulevard, 64111 Kansas City, MO USA

**Correction: BMC Medical Education (2024) 24:319**

10.1186/s12909-024-05292-7

Following publication of the original article [1], we have been informed that Figure 1 and 2 are switched.

The original article has been corrected.
