# Supplementary material for: Supporting underrepresented students in health sciences: a fuzzy cognitive mapping approach to program evaluation
Source: BMC Med Educ. 2024 Mar 20;24:319. doi: 10.1186/s12909-024-05292-7 (PMC10956253; doi:10.1186/s12909-024-05292-7)
Supplement: Supplementary file 1 — Additional file 1. STAHR Ambassador Map Code Book; the codebook used by researchers to code maps created by STAHR students. [file 12909_2024_5292_MOESM1_ESM.pdf]

Additional File 1. STAHR Ambassador Map Code Book

CODES	DEFINITION
FACILITATORS	
**Social Supports**	Having a support system inside and outside of program, spending time with family and friends, having a sense of connection and belonging. stress management programs,
**Career Development & Networking**	Networking opportunities with faculty, professionals in the field, etc.; Career development/advising (e.g. mock interviews, resume building);
**Mentorship (faculty & student mentors)**	Helpful mentorship (could be student of faculty mentor), Interactions with faculty; support from faculty or mentor.
**Motivation & Personal Factors**	Intrinsic motivation, self-motivation, goal-setting, attributes of the participant such as personal strengths or skills.
**Workshop/Meetings**	STAHR meetings, workshop topics, clusters, gatherings; online meetings
**Academics**	Passing exams, improved grades/test scores/ study skills/knowledge, doing well academically; academic preparation, taking certain courses, things related to coursework.
**Vulnerability/ Safe Environment**	Safe environment to express issues/concerns (e.g. asking questions; discussing mental health), self-expression, personal growth/development.
**Interdisciplinary**	Collaborations/interactions with students or faculty from other health science disciplines.
**Program Structure**	Program leadership (anything alluding to decisions of program), meeting diverse students with different ethnic backgrounds, program provides research and clinical experience, information gathered/distributed, STAHR scholarship; program flexibility (e.g. assignment dates); easy communication with STAHR teams/program; STAHR goodie bags
BARRIERS	
**Time**	Lack of time available, scheduling conflicts between STAHR and personal/school commitments; personal time constraints.
**Problematic Program Structure**	Lack of consistency in policies or practices, changes to STAHR required events, confusion on mentor/mentee roles, lack of diversity faculty/students, lack of experience (training and clinical activities), not flexible- related to STAHR program requirements (i.e. meeting schedules);

CODES	DEFINITION
	policies, practices, decisions made by STAHR program leadership.
**COVID-19/ remote learning**	Virtual learning due to COVID-19, zoom fatigue, isolation , internet/technical issues; curriculum, course, or experiences changes due to COVID-19
**Stressors/Outside Stressors-**	Personal life stressors, i.e., jobs, relationships, finances, distractions, lack of social support
**Lack of Engagement/ mentor issues**	Lack of 1:1 advising/mentorship, lack of networking opportunities, difficult to get ahold of mentor, issues w/ mentor relationships, individual experience with program engagement; (I.e. rude staff; unsupportive mentor; lack of informal discussions; unfriendly group)
**Self-Doubt/Academic preparedness**	Struggle w/ self-view, lack of personal motivation, not feeling academically prepared.
**Workshop/Meetings Barriers**	Repeating workshop topics (things purposefully set by STAHR staff), meetings for workshops that were not helpful/meaningful, lack new training topics; mismanagement of meeting times; rushed meetings; group too large during clusters
**Lack of Diversity/Equity/Inclusion**	Lack of diversity within health sciences programs, issues related to race/ethnicity, gender, SES, etc. (e.g. biases); being first generation college student;
