# Supplementary material for: Supporting underrepresented students in health sciences: a fuzzy cognitive mapping approach to program evaluation
Source: BMC Med Educ. 2024 Mar 20;24:319. doi: 10.1186/s12909-024-05292-7 (PMC10956253; doi:10.1186/s12909-024-05292-7)
Supplement: Supplementary file 2 — Additional file 2. Confirmatory Factor Analysis R Code.; the code used by the researcher to confirm the final two-factor structure of the concepts brought up in the maps by STAHR students. [file 12909_2024_5292_MOESM2_ESM.pdf]

Additional File 2. Confirmatory Factor Analysis R Code

```
model.syntax = "
    facilitator=~m1+m2+m4
    barrier=~m10+m12+m13+m14+m15
    "
model.fit = cfa(model.syntax, data=data, mimic="Mplus", estimator = "MLR")
#display model output
summary(model.fit, fit.measures = TRUE, standardized = TRUE)
```
